# APN1 is a functional receptor of Cry1Ac but not Cry2Ab in *Helicoverpa zea*

**DOI:** 10.1038/srep19179

**Published:** 2016-01-12

**Authors:** Jizhen Wei, Min Zhang, Gemei Liang, Kongming Wu, Yuyuan Guo, Xinzhi Ni, Xianchun Li

**Affiliations:** 1 State Key Laboratory for Biology of Plant Diseases and Insect Pests, Institute of Plant Protection, Chinese Academy of Agricultural Sciences, Beijing 100193, China; 2 Department of Entomology and BIO5 Institute, University of Arizona, Tucson, AZ 85721; 3 USDA-ARS, Crop Genetics and Breeding Research Unit, Tifton, GA 31793, USA

## Abstract

Lepidopteran midgut aminopeptidases N (APNs) are phylogenetically divided into eight clusters, designated as APN1–8. Although APN1 has been implicated as one of the receptors for Cry1Ac in several species, its potential role in the mode of action of Cry2Ab has not been functionally determined so far. To test whether APN1 also acts as one of the receptors for Cry1Ac in *Helicoverpa zea* and even for Cry2Ab in this species, we conducted a gain of function analysis by heterologously expressing *H. zea* APN1 (*Hz*APN1) in the midgut and fat body cell lines of *H. zea* and the ovarian cell line of *Spodoptera frugiperda* (Sf9) and a loss of function analysis by RNAi (RNA interference) silencing of the endogenous APN1 in the three cell lines using the *HzAPN1* double strand RNA (dsRNA). Heterologous expression of *HzAPN1* significantly increased the susceptibility of the three cell lines to Cry1Ac, but had no effects on their susceptibility to Cry2Ab. Knocking down of the endogenous APN1 made the three cell lines resistant to Cry1Ac, but didn’t change cell lines susceptibility to Cry2Ab. The findings from this study demonstrate that HzAPN1 is a functional receptor of Cry1Ac, but not Cry2Ab.

Aminopeptidases N (APNs) are a class of broad-specificity metalloaminopeptidases that sequentially and preferentially remove neutral amino acids from the N-terminus of a range of peptides in microorganisms, plants and animals^1^. They share two conserved signature motifs: a catalytic zinc binding/gluzincin motif HEXXH-(X18)-E and an N-terminal amino acid substrate-binding exopeptidase motif GXMEN^2^,^3^. In the catalytic HEXXH-(X18)-E motif, the two histidine residues and the distant gluatamic acid residue constitute zinc ligands, whereas the gluatamic acid residue between the two histidine residues and zinc ion catalyze the sequential release of N-terminal neutral amino acids^3^. By virtue of the two conserved motifs, APNs efficiently retrieve amino acids from dietary proteins and endogenous proteins degraded during protein turnover and thus play important roles in nutrition, protein maturation, peptide hormone level regulation, stress response, cell signaling, cell cycle control, and cellular processes involved in health and diseases^1^,^4^.

In recent years, lepidopteran midgut APNs are widely studied because of their potential role as one of the receptors for the *Bacillus thuringiensis* (Bt) crystal insecticidal toxins (Cry toxins)^5^,^6^, which are used extensively in sprays and transgenic crops to control lepidopteran insect pests^7^,^8^. Lepidopteran midgut APNs are 100 to 180 kDa and mainly bound to microvillar membranes of midgut cells via a C-terminal glycosyl phosphatidyl inositol (GPI) anchor. Over 140 APN cDNAs have been cloned from more than 20 lepidopteran species (http://www.ncbi.nlm.nih.gov/). Phylogenetic analysis has resolved them into 8 clusters, designated as APN1–8 respectively, which are derived from multiple gene duplications^9^,^10^. Each lepidopteran species usually has no more than one APN for each cluster and their amino acid sequence identity ranges from 23 to 40% between different clusters of the same species and from 50 to 95% between members of the same cluster in different species^9^.

Although Cry1Ac and Cry2Ab are two of the most widely used Bt Cry toxins in transgenic crops^8^,^11^, studies on defining the role of insect APNs as a receptor for Cry toxins have been mostly focused on Cry1Ac. Among the 8 clusters of lepidopteran APNs, there is no evidence for involvement of APN4, APN7 and APN8 as a receptor for Cry1Ac, Cry2Ab, or other Cry toxins^12^,^13^,^14^. Conflicting evidence exists for APN2, because 1) it can bind to Cry1Ac in *Helicoverpa armigera*^15^, but not in *Lymantria dispar*^16^; and 2) it is not linked to Cry1Ac resistance in *Plutella xylostella*^17^. APN3 and APN5 can bind to Cry1Ac in *H. armigera*^18^ and *P. xylostella*^17^ respectively, but their involvement in Cry1Ac resistance has not been documented. APN6 seems not to be a receptor for Cry1Ac and Cry2Ab because it can not bind to Cry1Ac in *H. armigera*^19^ and is not involved in Cry1Ac and Cry2Ab resistance in *Trichoplusia ni*^14^,^20^. In stark contrast, various studies with APN1 from *Manduca sexta*^21^, *L. dispar*^22^,^23^, *P. xylostella*^24^, *H. armigera*^15^,^25^, and *T. ni*^20^ consistently demonstrate that it is one of the midgut receptors for Cry1Ac.

So far, the only study examined APN’s role in the mode of action of Cry2Ab shows that APN5 can bind to Cry2Ab in *P. xylostella*^26^. Whether APN1 also serves as one of the receptors for Cry2Ab still remains unclear. Presence of cross-resistance between Cry1Ac and Cry2Ab^27^,^28^,^29^ and detection of a Cry1Ac- and Cry2Ab-binding protein with a similar size (120 ~ 130 kDa) of APN1 in the midgut and fat body cell lines of *H. zea* by ligand blot suggest a possibility of APN1 as a shared receptor for Cry2Ab and Cry1Ac (Wei & Li, unpublished manuscript). To test this hypothesis, we conducted a gain of function analysis by heterologously expressing *H. zea* APN1 (*Hz*APN1) in the midgut and fat body cell lines of *H. zea* and the ovarian cell line of *Spodoptera frugiperda* (Sf9) and a loss of function analysis by knocking down the endogenous APN1 in the three cell lines using the *HzAPN1* double strand RNA (dsRNA). Heterologous expression of *HzAPN1* significantly enhanced the susceptibility of the three cell lines to Cry1Ac, but not to Cry2Ab. Knocking down of the endogenous APN1 made the three cell lines more tolerant to Cry1Ac, but didn’t change their susceptibility to Cry2Ab. The data demonstrate that APN1 is a receptor for Cry1Ac, but not for Cry2Ab.

## Results

### Binding profiles of Cry1Ac and Cry2Ab in *H. zea* larval midgut BBMV

**Figure 1:**
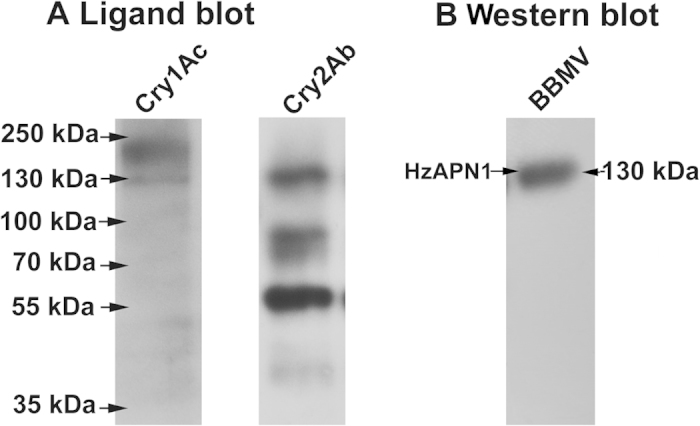
Ligand blot detection of Cry1Ac and Cry2Ab binding proteins in *H. zea* larval midgut BBMV (**A**) and Western blot detection of HzAPN1 in *H. zea* larval midgut BBMV (**B**).

As in *H. zea* midgut and fat body cell lines (Wei & Li, unpublished manuscript), ligand blot detected two binding bands of 210 and 130 kDa for Cry1Ac (Fig. 1A, left panel) and four binding proteins of 130 and 87, 57 and 42 kDa for Cry2Ab (Fig. 1A, right panel) in the brush border membrane vesicles (BBMV) isolated from *H. zea* larval midguts. Western blot analysis of *H. zea* larval midgut BBMV (Fig. 1B) with the antibody directed against APN1 from *H. armigera*^30^, a sister species of *H. zea*, revealed that the common binding band of 130 kDa by Cry1Ac and Cry2Ab was co-localized with *H. zea* APN1.

### Impact of heterologous expression of HzAPN1 on cytotoxicities of Cry1Ac and Cry2Ab

**Figure 2:**
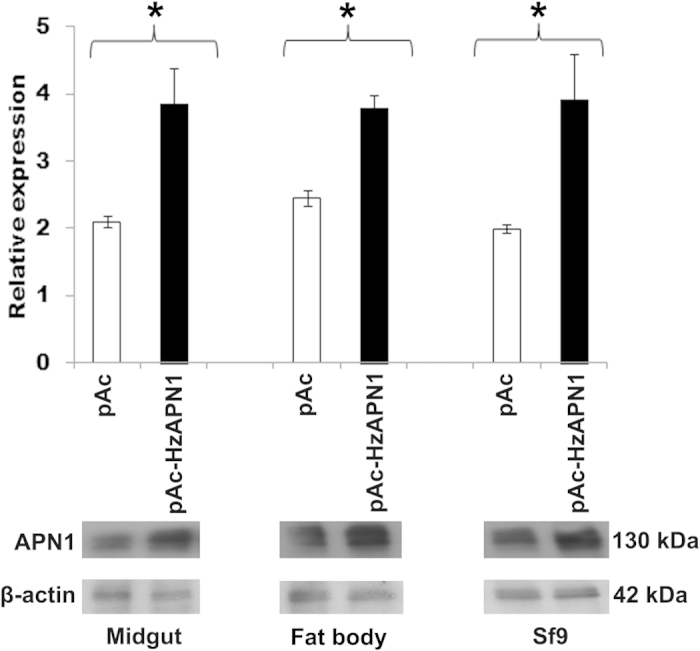
Expression level of APN1 in the cells transfected with pAc or pAc-HzAPN1 plasmids. For pAc (empty vector control) and pAc-HzAPN1 plasmids, three independent transfections were done for each cell line and the protein extracts from each of the three transfections were analyzed by Western blot. The lower panel picture of HzAPN1 and β-actin bands is a representative of the three Western blots. The average expression of HzAPN1 relative to that of β-actin calculated by Image J quantification of the three Western blots is showed in the upper panel. Each error bar represents the standard error of the mean from three transfection replicates. Significant differences in relative expression of HzAPN1 between pAc and pAc-HzAPN1 transfected cells of each cell line are indicated with an asterisk (*) (*P* < 0.05, Student’s t-test).

Western blot showed that Sf9 cells and *H. zea* midgut and fat body cells transfected with pAc-HzAPN1 produced significantly more APN1 proteins than the three cell lines transfected with the empty vector pAc (control cells) (Fig. 2; *P*_*midgut*_ = 0.035*, P*_*fat body*_ = 0.004 *and P*_*Sf9*_ = 0.048). More precisely, the protein level of APN1 in the pAc-HzAPN1 transfected midgut, fat body and Sf9 cells was 1.83, 1.55 and 1.97 times more than that in the corresponding pAc-transfected control cells.

When the above pAc- and pAc-HzAPN1-transfected cells were exposed to 15 μg/ml of the activated Cry1Ac, mortality increased from none (−2.64%) for pAc-transfected Sf9 cells to 48.05% for pAc-HzAPN1 transfected Sf9 cells (Table 1). Consistent with the higher APN1 increase in the pAc-HzAPN1 transfected midgut cells than in the pAc-HzAPN1 transfected fat body cells (Fig. 1), the mortality of the former increased by 55.95%, whereas it was 20.90% for the latter. When the concentration of activated Cry1Ac was doubled, mortality was increased significantly (for at least two to three times) from 24.19 (pAc control) to 76.17% (pAc-HzAPN1) for midgut cells, 23.77 to 51.57% for fat body cells and 10.61 to 44.35% for Sf9 cells, respectively (Table 1). When the pAc and pAc-HzAPN1 transfected cells were treated with 2.5 or 3.75 μg/ml of the activated Cry2Ab, no significant mortality enhancement by heterologous expression of HzAPN1 was observed regardless of cell line and Cry2Ab concentration (Table 1).

### Effect of knocking down APN1 on cytotoxicities of Cry1Ac and Cry2Ab

**Figure 3:**
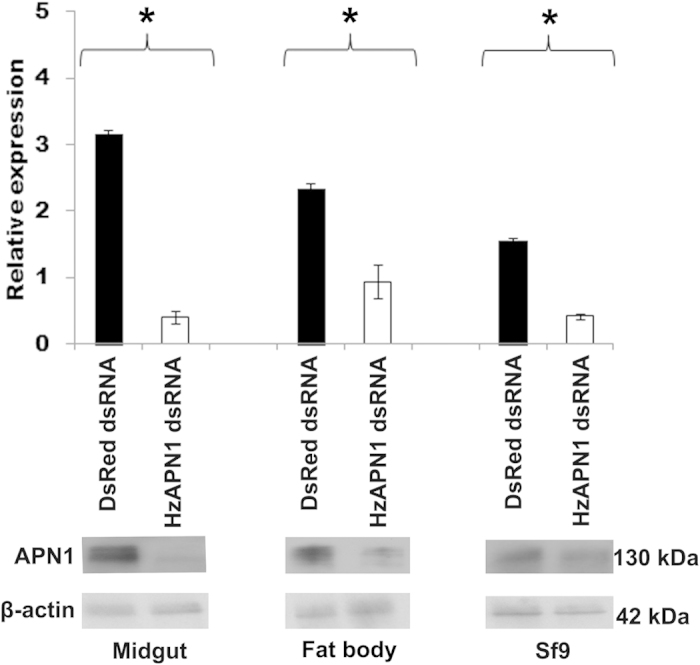
Expression level of APN1 in the cells transfected with DsRed or HzAPN1 dsRNA. For DsRed (dsRNA control) and HzAPN1 dsRNA, three independent transfections were conducted for each cell line and the protein extracts from each of the three transfections were analyzed by Western blot. The lower panel picture of APN1 and β-actin bands is a representative of the three Western blots. The average expression of HzAPN1 or SfAPN1 (Sf9 cells) relative to that of β-actin calculated by Image J quantification of the three Western blots is displayed in the upper panel. Each error bar represents the standard error of the mean from three transfection replicates. Significant differences in relative expression of HzAPN1 or SfAPN1 (Sf9 cells) between DsRed and HzAPN1 dsRNA transfected cells of each cell line are indicated with an asterisk (*) (*P* < 0.05, Student’s t-tests).

Western blot detected 7.87-fold reduction in the protein level of APN1 in *H. zea* midgut cells transfected with 50 nM HzAPN1 dsRNA, compared with *H. zea* midgut cells transfected with 50 nM DsRed dsRNA (control cells; *P*_*midgut*_ < 0.0001) (Fig. 3). Likewise, transfection of 50 nM HzAPN1 dsRNA also resulted in 2.47- (*P*_*fat body*_  = 0.0098) and 3.74-fold (*P*_*Sf9*_ < 0.0001) decrease in the protein level of the endogenous APN1 in *H. zea* fat body cells and Sf9 cells, respectively (Fig. 3).

When the above HzAPN1 dsRNA-transfected *H. zea* midgut cells were exposed to 150 μg/ml of the activated Cry1Ac, a 33.79% decrease in mortality was exhibited when compared with that of the DsRed dsRNA-transfected *H. zea* midgut cells (Table 2). Due to the lower knocking down activities of the endogenous APN1 in the other two cell lines (Fig. 3), HzAPN1 dsRNA only produced a 19.29% and a 13.12% reduction in mortality for *H. zea* fat body cells and Sf9 cells, respectively (Table 2). In contrast, no significant mortality reduction by knocking down of the endogenous APN1 was seen in the three insect cell lines when they were treated with 30 μg/ml of the activated Cry2Ab (Table 2).

## Discussion

Both Cry1Ac and Cry2Ab are expected to have two or more receptors in the larval midguts of Lepidopteran insects (Wei & Li, unpublished manuscript). Documentation of cross-resistance between the two toxins implies that they may share one common receptor^27^,^28^,^29^, although cross-resistance can occur through receptors-independent mechanisms. Recognition of one protein band with the same size of APN1 (120 ~ 130 kDa) by both Cry1Ac and Cry2Ab antibodies in the midgut and fat body cell lines of *H. zea* (Wei & Li, unpublished manuscript) and by Cry2Aa antibody in *Spodoptera littoralis* BBMV^31^ suggest that the potential common receptor could be APN1. This present study was conducted to test whether this hypothesis is true or not.

By ligand blot and Western blot, we confirmed that *H. zea* larval midgut BBMV, like *H. zea* midgut and fat body cell lines (Wei & Li, unpublished manuscript), also had a 130 kDa protein band that was recognized by Cry1Ac, Cry2Ab, and APN1 antibodies (Fig. 1). Heterlogous expression of HzAPN1 significantly increased the susceptibilities of Sf9, *H. zea* midgut and fat body cell lines to activated Cry1Ac (Fig. 2 and Table 1), whereas RNAi knocking down of the endogenous APN1 made the three cell lines significantly less susceptible to activated Cry1Ac (Fig. 3 and Table 2). In contrast, neither heterologous expression of HzAPN1 nor RNAi knocking down of the endogenous APN1 affected the susceptibilities of the three cell lines to activated Cry2Ab (Tables 1 and 2). The data suggest that the 130 kDa band recognized by Cry1Ac antibody is HzAPN1, but the 130 kDa band recognized by Cry2Ab is an unknown protein. Moreover, the data directly demonstrate that HzAPN1 is one of the receptors for Cry1Ac, but not for Cry2Ab in *H. zea*.

While APN1 has been implicated as one of the receptors for Cry1Ac in a number of species^15^,^21^,^23^, this is the first report that functionally characterized APN1 as a receptor of Cry1Ac in *H. zea*. This is consistent with the result of Sivakumar *et al*., who confirmed that APN1 from *H. armigera* (HaAPN1)^32^, the closest orthologue of HzAPN1, acted as a receptor of Cry1Ac in Sf21 cells. To our knowledge, the current study is also the first report that experimentally excluded APN1 as the basis of cross-resistance between the two toxins—the common receptor of Cry1Ac and Cry2Ab. However, this study cannot be extrapolated to rule out the possibility of other APNs as one of the receptors for Cry2Ab. First, the size of the protein band (120 ~ 130 kDa) recognized by Cry2Ab antibody not only matches with the size of APN1, but also falls into the size range of other APNs. In addition, APN5 from *P. xylostella* (PxAPN5) was reported to bind to Cry2Ab^26^. Further functional analyses of HzAPN5 and other APNs from *H. zea* are needed to resolve this issue.

The fact that RNAi knocking down of the endogenous APN1 protected not only midgut cells but also fat body and ovarian (Sf9) cells from the toxic effects of Cry1Ac (Fig. 3 and Table 2) suggests that APN1 may be expressed in both midgut and non-gut hemocoelic tissues at least in some species. Consistent with this notion, APN1 has been shown to be expressed in midgut, malpighian tubule, salivary gland and fat body in *T. ni*^33^, fat body, malpighian tubule and salivary gland in *Achaea janata*^34^, midgut, fat body, malpighian tubule, epidermis and hemolymph in *S. exigua*^35^, midgut, fat body, Malpighian tubule and carcass in *Epiphyas postvittana*^36^, and midgut and Malpighian tubule in *B. mori*^37^. The toxic effects of Cry1Ac and Cry2Ab on *in vitro* cultured fat body and ovarian (Sf9) cells observed in this study and Wei *et al.* (Wei & Li, unpublished manuscript) as well as the effects of intrahemocoelic injections of several Cry1 toxins on the growth and survival of *Lymantria dispar* and *Neobellieria bullata* larvae^38^ and of *A. janata* larvae^34^ further strengthen the notion of APNs expression in the above non-gut hemocoelic tissues. Apparently, these receptors-expressing non-gut hemocoelic tissues can serve as additional *in vivo* target tissues of Cry toxins, as long as these toxins can cross the insect midgut epithelium and reach the haemolymph when fed orally. Detection of small amounts of orally ingested Cry toxins in the haemolymph of *Lygus Hesperus*^39^ proves that Cry toxins can naturally cross the insect gut into the haemolymph at least in some species, but probably at a very low speed. Novel protein conjugation/fusion technology capable of speeding up the crossing of the haemocoelic-active Cry toxins across the insect gut into haemolymph is needed to further enhance the efficacy of Cry toxins by virtue of targeting both midgut and non-gut hemocoelic tissues.

## Methods

### Insects and insect cell lines

Three insect cell lines and one susceptible strain of *H. zea* were used in this study. The *H. zea* susceptible strain was established with a few thousand eggs purchased from Benzon Research Inc. (Carlisle, PA) in October 2014 and maintained on wheat germ-containing diets at 27 ± 1 °C, 60 ± 10% RH and a photoperiod of 14 L:10 D^40^. The *H. zea* midgut cell line RP-HzGUT-AW1(MG) and *H. zea* fat body cell line BCIRL-HzFB33(FB), generously provided by Dr. Cynthia L.Goodman (BCIRL, USDA, ARS)^41^, were routinely maintained with Excell 420 insect serum-free medium (SAFC Bioscience, Lenexa, KS) supplemented with 10% heat-inactivated fetal bovine serum (FBS, Hyclone-QB perbio, Logan, UT), 50 U/ml penicillin, 50 mg/ml streptomycin, and 12 mg/ml gentamycin (Invitrogen, CA) in an incubator at 28 °C. Sf9 cell line derived from pupal ovarian tissue of *Spodoptera frugiperda* was cultured in Sf-900 II SFM medium (GIBCO/BRL/Life Technologies) supplemented with 10% heat-inactivated fetal bovine serum, 50 U/ml penicillin, 50 mg/ml streptomycin, and 12 mg/ml gentamycin in the same incubator.

### Toxins and antibody

Cry1Ac and Cry2Ab protoxin were supplied by Biotechnology Research Laboratory, Institute of Plant Protection, Chinese Academy of Agricultural Sciences. Activated Cry1Ac and Cry2Ab were prepared from the corresponding protoxin as described by Wei *et al.* (Wei & Li, unpublished manuscript). Anti-Cry1Ac antibody and anti-HaAPN1 antibody were obtained from Dr. Chenxi Liu of the Institute of Plant Protection, Chinese Academy of Agricultural Sciences^30^. Preparation of anti-Cry2Ab antibody was described in Wei *et al.* (Wei & Li, unpublished manuscript).

### Ligand blot detection of CryAc and Cry2Ab binding proteins in *H. zea* larval midgut BBMV

Brush border membrane vesicles (BBMV) for ligand blot and Western blot (see below) were prepared from the 5^th^ instar larvae midguts of the susceptible strain of *H. zea* using a differential centrifugation method^42^,^43^. When dissected out, midguts were open to remove peritrophic membranes and gut contents, cleaned in ice-cold 0.7% NaCl solution, placed on filter paper for a few seconds to remove excessive water, weighed and stored at −80 °C. The frozen midguts taken out from the freezer were homogenized in nine-fold volume (w/v) of ice-cold buffer A (300 mM mannitol, 5 mM EGTA, 17 mM Tris-HCl, 1 mM PMSF), incubated with one volume of 24 mM MgCl_2_ on ice for 15 min, and centrifuged at 2500 × *g* and 4 °C for 15 min. The supernatant was centrifuged at 30,000 × *g* and 4 °C for 30 min. The resulting pellet was suspended in buffer B (150 mM Mannitol, 2.5 mM EGTA, 8.5 mM Tris-HCl, 1 mM PMSF), left on ice up to 4 h, and then centrifuged at 30,000 *g* and 4 °C for 15 min. The final pellet was suspended in buffer C (150 mM NaCl, 5 mM EGTA, 1 mM PMSF, 20 mM Tris-HCl, 1% CHAPS) and used as the BBMV preparation. The protein concentrations of the BBMV preparations were determined by the Bradford protein assay, using bovine serum albumin (BSA) as a standard (Bradford, 1976). When conducted ligand blot, 8 μg protein of the larval midgut BBMV preparation was separated on a 10% SDS-PAGE gel and electroblotted to a polyvinylidene difluoride (PVDF) membrane (Thermo scientific) in the transfer buffer (25 mM Tris, 192 mM glycine, 10% methanol, pH 8.3). The follow-up procedures for detection of Cry1Ac or Cry2Ab binding proteins using the anti-Cry1Ac antibody or anti-Cry2Ab antibody were the same as described in Wei *et al.* (Wei & Li, unpublished manuscript).

### Western blot detection of APN1 in larval midgut BBMV and cell line protein extracts

The larval midgut BBMV was prepared as above and the protein extracts of the three insect cell lines transfected with the control plasmid pAc, pAc-HzAPN1, DsRed double strand RNA (dsRNA), or HzAPN1 dsRNA (see preparation and transfection of pAc-HzAPN1 plasmid and dsRNA below) were prepared as described by Wei *et al.* (Wei & Li, unpublished manuscript). Six μg protein of the larval midgut BBMV and/or the protein extract of each cell line transfected with pAc, pAc-HzAPN1, DsRed dsRNA, or HzAPN1 dsRNA were separated on 10% sodium dodecyl sulfate (SDS)-polyacrylamide gel electrophoresis (PAGE) gel and electroblotted to a polyvinylidene difluoride (PVDF) membrane (Thermo scientific) in the transfer buffer (25 mM Tris, 192 mM glycine, 10% methanol, pH 8.3). The membrane was blocked with phosphate buffered saline (PBS; 137 mM NaCl, 2.7 mM KCl, 10 mM Na_2_HPO_4_, 2 mM KH_2_PO_4_, pH 7.4) containing 0.1% Tween 20 (Bio-Rad) (PBST buffer) and 5% milk (Amresco) at 4 °C overnight or room temperature for at least 2 h. The membrane was then hybridized at 4 °C with the primary antibody anti-HaAPN1 antiserum (adding to the blocking buffer at 1:15,000 dilution) for about 14 h, washed three times of 15 min each with PBST buffer, probed at 4 °C with the horseradish peroxidase (HRP)-conjugated secondary antibody (ZSGB-BIO, China) (1:15,000) for 2 h, washed three times of 15 min each with PBST buffer, and visualized with immobilon western chemiluminescent HRP substrate (Millipore Corporation, Billerica, MA, USA)^30^.

A duplicate SDS-PAGE gel of the larval midgut BBMV and/or cell line protein extracts was electrophoresed simultaneously to detect the reference protein β-actin. The procedure for detection of β-actin was identical to that for APN1 except that the membrane blot was hybridized with anti-β-actin antibody (sc-69879, Santa Cruz Biotechnology, USA) (1:3000) for 2 h at room temperature with constant shaking and then probed with a goat anti-mouse IgM-HRP (Santa Cruz Biotechnology, USA) (1:3000) for 1 h at room temperature with constant shaking.

### Construction of pAC-HzAPN1 construct

The open reading frame (ORF) of HzAPN1 was PCR-amplified from the plasmid HzAPN1-pGEMT (Zhang & Li, unpublished manuscript) with the gene-specific primers APN-5′ASCI (5′-GGCGCGCCGACCAGCGTGGAGTCACAA-3′) and APN-3′NOTI (5′-GCGGCCGCTTAAGCCATATTAACAACGAGAGTCA-3′). PCR reaction (20 μL) contained 1 μL of HzAPN1-pGEMT (Zhang & Li, unpublished manuscript), 1 μL of LongAmp® Taq DNA polymerase (New England Biolabs, Ipswich, MA), 1.6 μL of dNTP (5 mM), 2 μL of primer mix (25 mM each), 4 μL of 5× longAmp Taq reaction buffer and 10.4 μL ddH_2_O. The PCR reaction was initiated at 94 °C for 5 min, followed by 30 cycles of 94 °C for 30 s, 60 °C for 30 s and 65 °C for 1 min, and a final extension of 5 min at 65 °C. The PCR products were TA-cloned into pGEM®-T vector (Promega, Madison, WI), released by double digestion with ASCI and NOTI (New England Biolabs, Ipswich, MA), and subcloned into the expression vector pAc/V5-His A (Invitrogen) via the ASCI and NOTI enzyme sites.

### Production of HzAPN1 and DsRed dsRNA

A 658 bp cDNA sequence of HzAPN1 was PCR-amplified from HzAPN1-pGEMT (Zhang & Li, unpublished manuscript) using the primers HZAPN1 dsRNA-F (5′-GGATCCTGT CTGACTCCCTTGACTCTGCTC-3′) and HzAPN1 dsRNA-R (5′-CGGTTGTATTC GTGGATTGATAGCCT-3′). The PCR reaction (20 μL) was composed of 1 μL of HzAPN1-PGEMT, 1 μL of LongAmp® Taq DNA polymerase (New England Biolabs, Ipswich, MA), 1.6 μL of dNTP (5 mM), 2 μL of primer mix (25 mM each), 4 μL of 5× longAmp Taq reaction buffer and 10.4 μL ddH_2_O. The PCR conditions were 94 °C for 5 min, followed by 30 cycles of 94 °C for 30 s, 56 °C for 30 s and 65 °C for 1 min, and a final extension of 5 min at 65 °C. The resultant PCR product was TA-cloned into the pGEM-T Easy-vector to generate the template plasmid HzAPN1-pGEMT for production of HzAPN1 dsRNA. Likewise, a 480-bp cDNA sequence of DsRed was PCR-amplified from pBac [3 × P3-DesRedaf] with the primers DsRED-F (TGCAGGTGACCAAGGGC) and DsRED-R (CGTTGTGGGAGGTGATGT) and TA-cloned into the pGEM-T Easy-vector to generate the template plasmid DsRed-pGEMT for production of the DsRed dsRNA (i.e. control dsRNA).

We used two pairs of universal primers complementary to the flanking sequence of the multiple cloning sites of the pGEM-T Easy-vector to PCR-amplify two *in vitro* transcription templates (one for sense RNA, one for antisense RNA) from the template plasmids DsRed-pGEMT and HzAPN1-pGEMT, respectively. The primer pair T7 pGEMTeasy Fwd (5′-GGTGTAATACGACTCACTATAGGG-3′) and pGEMTeasy Rev (5′-CAAGCTATGCATCCAACGCGTTGGGAG-3′) were used to PCR-amplify the sense template that had a T7 RNA polymerase promoter sequence at the 5′ end of its sense strand. Another primer pair T7 pGEMTeasy Rev (5′-GGTGTAATACGACTCACTATAGGGCAAGCTATGCATCCAACGCGTTGGGAG-3′) and pGEMTeasy Fwd (5′-CGAATTGGGCCCGGACGTCGCA-3′) were used to PCR-amplify the antisense template that had a T7 RNA polymerase promoter sequence at the 5′ end of its antisense strand. The resulting sense and antisense template PCR products were cleaned, quantified, and combined at 1:1 ratio. Then the combined sense and antisense templates were used to make dsRNA by simultaneous *in vitro* transcription of both the sense and antisense RNA using the RiboMax Large Scale RNA Production System (Promega) according to the product manual. Following *in vitro* transcription, the DsRed and HzAPN1 dsRNA products were cleaned with MEGAclear Kit (Ambion Inc.), quantified and stored at −80 °C for subsequent transfection.

### Cell transfection and toxicity bioassay

*H. zea* midgut, fat body and Sf9 cells were seeded onto a 12-well plate (9 × 10^5 ^cells/well) and incubated at 28 °C, respectively. For heterologous expression of HzAPN1 in the three cell lines, cells were transfected with 2 μg/well of pAc (control, empty vector) or pAC-HzAPN1 plasmids using Cellfectin (Invitrogen; 8 μl per well) for 5 h. For RNA interference (RNAi) silencing of endogenous APN1 in the three cell lines, cells were transfected with 50 nM of DsRed (control) or HzAPN1 dsRNA for 5 h. We then replaced the transfection mixture of each well with 1.5 ml of supplemented Excell 420 (midgut and fat body cells) or Sf-900 II SFM medium (Sf9 cells) and incubated the cells at 28 °C for 64 hours. The cells in each independent transfection replicate were reseeded onto three wells in a 96-well micro-plate at 10000 cells in 100 μL per well. After 2 h of attachment, the cells were treated with activated Cry1Ac or activated Cxry2Ab. The concentrations for bioassays of the cells transfected with pAc (control) or pAc-HzAPN1 plasmids were 15 and 30 μg/ml for activated Cry1Ac and 2.5 and 3.75 and μg/ml for activated Cry2Ab), respectively. The concentrations for bioassays of the cells transfected with DsRed dsRNA (control) and HzAPN1 dsRNA were 150 μg/ml for activated Cry1Ac and 30 μg/ml for activated Cry2Ab, respectively. Cell mortality was determined by replacing 35 μL medium from each well with 35 μL of 0.4% trypan blue (AMRESCO^®^, BioExpress, Ohio, USA) and counting the numbers of stained (dead cells) and unstained (live cells) in two 400x fields of view from the central parts of each well under an inverted microscope (VistaVision, VWR) after 4.5 h for Cry1Ac or 5.5 hours for Cry2Ab(Wei & Li, unpublished manuscript). Each plasmid or dsRNA was independently transfected into each cell three times and each independent transfection was bioassayed three times with control or a given concentration of Cry1Ac or Cry2Ab (3 × 3 = 9 replicates).

## Statistical Analysis

Cell mortality was calculated by dividing the number of dead cells (the number of cells before toxin treatment – the number of unstained cells after treatment) by the number of cells before treatment and corrected with the Abbott formula^44^. Significant differences in mortality between the control plasmid pAc (or the DsRed control dsRNA) and pAc-HzAPN1 (or HzAPN1 dsRNA) were compared using Student t-test with α = 0.05 (JMP 8; SAS Institue, Inc.). Mortality values of different treatments were arcsine transformed before analysis.

The band intensities of the target protein APN1 and the reference protein β-actin on all the Western blot pictures of the protein extracts from the three cell lines transfected with pAc, pAc-HzAPN1, DsRed dsRNA, or HzAPN1 dsRNA were quantified by densitometry using Image J software (NIH, v1.46). The relative expression level of APN1 in each control or treatment cells was calculated by dividing the band intensity of APN1 by the band intensity of β-actin. Significant differences in the relative expression of APN1 between the control plasmid pAc (or the DsRed control dsRNA) and pAc-HzAPN1 (or HzAPN1 dsRNA) were evaluated by Student’s t-test (JMP 8; SAS Institue, Inc.).

## Additional Information

**How to cite this article**: Wei, J. *et al.* APN1 is a functional receptor of Cry1Ac but not Cry2Ab in *Helicoverpa zea.* *Sci. Rep.* **6**, 19179; doi: 10.1038/srep19179 (2016).

## Figures and Tables

**Table 1:** Cytotoxcity of activated Cry1Ac and Cry2Ab to *H. zea* midgut, fat body and Sf9 cell lines transfected with pAc or pAc-HzAPN1 plasmids*.

Cell lines	Treatments & *P* values	Cry1Ac (30 μg/ml)	Cry1Ac (15 μg/ml)	Cry2Ab (3.75 μg/ml)	Cry2Ab (2.5 μg/ml)
Midgut cells	pAc	24.19 ± 3.45	15.43 ± 1.93	56.25 ± 9.79	28.01 ± 3.98
	pAc-HzAPN1	76.17 ± 3.57	71.38 ± 3.58	55.19 ± 1.99	31.91 ± 7.92
	*P*	0.0005	0.0002	0.9214	0.8866
Fat body cells	pAc	23.77 ± 3.32	14.46 ± 1.76	62.75 ± 3.44	34.81 ± 1.07
	pAc-HzAPN1	51.57 ± 6.63	35.36 ± 6.81	55.80 ± 1.79	34.5 ± 3.86
	*P*	0.02	0.041	0.1474	0.8087
Sf9 cells	pAc	10.61 ± 0.77	−2.64 ± 6.19	50.41 ± 4.45	20.22 ± 5.71
	pAc-HzAPN1	44.35 ± 5.78	48.05 ± 6.57	57.86 ± 1.29	17.94 ± 7.18
	*P*	0.0044	<0.0001	0.1828	0.8165

*Values in the table are average mortality ± standard errors of three replicates of three independent transfections of each plasmid per cell line. Mortality values of different treatments were arcsine transformed before analysis. *P* values refer to the morality comparisons between pAc and pAc-HzAPN1 transfected cells of each cell line (Student’s t-test in JMP 8; SAS Institute, Inc.).

**Table 2:** Cytotoxcity of activated Cry1Ac and Cry2Ab to *H. zea* midgut, fat body and Sf9 cell lines transfected with DsRed or HzAPN1 dsRNA.

Cell lines	Treatments & P values	Cry1Ac (150 μg/ml)	Cry2Ab (30 μg/ml)
Midgut cells	DsRed dsRNA	81.70 ± 1.85	86.19 ± 1.31
	HzAPN1 dsRNA	47.91 ± 0.92	85.21 ± 4.11
	*P*	<0.0001	0.831
Fat body cells	DsRed dsRNA	87.38 ± 1.56	83.94 ± 2.50
	HzAPN1 dsRNA	68.09 ± 2.89	80.71 ± 3.24
	*P*	0.0042	0.4735
Sf9 cells	DsRed dsRNA	61.20 ± 3.37	45.79 ± 2.90
	HzAPN1 dsRNA	48.08 ± 3.24	48.86 ± 2.15
	*P*	0.0486	0.4428

*Values in the table are average mortality ± standard errors of three replicates of three independent transfections of each dsRNA per cell line. Mortality values of different treatments were arcsine transformed before analysis. *P* values refer to the morality comparisons between DsRed and HzAPN1 dsRNA transfected cells of each cell line (Student’s t-test in JMP 8; SAS Institute, Inc.).
